# High-density genetic mapping of Fusarium head blight resistance and agronomic traits in spring wheat

**DOI:** 10.3389/fpls.2023.1134132

**Published:** 2023-05-10

**Authors:** Samia Berraies, Richard Cuthbert, Ron Knox, Arti Singh, Ron DePauw, Yuefeng Ruan, Firdissa Bokore, Maria Antonia Henriquez, Santosh Kumar, Andrew Burt, Curtis Pozniak, Amidou N’Diaye, Brad Meyer

**Affiliations:** ^1^ Swift Current Research and Development Center, Agriculture and Agri-Food Canada, Swift Current, SK, Canada; ^2^ Department of Agronomy, Iowa State University, Ames, IA, United States; ^3^ Retired, Calgary, AB, Canada; ^4^ Morden Research and Development Centre, Agriculture and Agri-Food Canada, Morden, MB, Canada; ^5^ Brandon Research and Development Centre, Agriculture and Agri-Food Canada, Brandon, MB, Canada; ^6^ Ottawa Research and Development Centre, Agriculture and Agri-Food Canada, Ottawa, ON, Canada; ^7^ Department of Plant Sciences, University of Saskatchewan, Saskatoon, SK, Canada

**Keywords:** *Triticum aestivum*, Fusarium head blight, quantitative trait loci, genetic resistance, molecular breeding

## Abstract

Fusarium head blight (FHB) has rapidly become a major challenge to successful wheat production and competitive end-use quality in western Canada. Continuous effort is required to develop germplasm with improved FHB resistance and understand how to incorporate the material into crossing schemes for marker-assisted selection and genomic selection. The aim of this study was to map quantitative trait loci (QTL) responsible for the expression of FHB resistance in two adapted cultivars and to evaluate their co-localization with plant height, days to maturity, days to heading, and awnedness. A large doubled haploid population of 775 lines developed from cultivars Carberry and AC Cadillac was assessed for FHB incidence and severity in nurseries near Portage la Prairie, Brandon, and Morden in different years, and for plant height, awnedness, days to heading, and days to maturity near Swift Current. An initial linkage map using a subset of 261 lines was constructed using 634 polymorphic DArT and SSR markers. QTL analysis revealed five resistance QTL on chromosomes 2A, 3B (two loci), 4B, and 5A. A second genetic map with increased marker density was constructed using the Infinium iSelect 90k SNP wheat array in addition to the previous DArT and SSR markers, which revealed two additional QTL on 6A and 6D. The complete population was genotyped, and a total of 6,806 Infinium iSelect 90k SNP polymorphic markers were used to identify 17 putative resistance QTL on 14 different chromosomes. As with the smaller population size and fewer markers, large-effect QTL were detected on 3B, 4B, and 5A that were consistently expressed across environments. FHB resistance QTL were co-localized with plant height QTL on chromosomes 4B, 6D, and 7D; days to heading on 2B, 3A, 4A, 4B, and 5A; and maturity on 3A, 4B, and 7D. A major QTL for awnedness was identified as being associated with FHB resistance on chromosome 5A. Nine small-effect QTL were not associated with any of the agronomic traits, whereas 13 QTL that were associated with agronomic traits did not co-localize with any of the FHB traits. There is an opportunity to select for improved FHB resistance within adapted cultivars by using markers associated with complementary QTL.

## Introduction

The southern regions of the Prairie Provinces of Saskatchewan, Alberta, and Manitoba are the largest spring wheat growing areas, producing about 98% of all Canadian spring wheat (2017 Canadian Wheat Crop in Review). The focus of the wheat breeding programs in most of these regions is Fusarium head blight (FHB) disease, to which wheat is particularly susceptible. The infection by FHB leads to reduced grain yield *via* kernel weight and size, poor milling and baking quality, and reduced germination ability. Furthermore, *Fusarium graminearum*, the causal agent of FHB, produces the mycotoxin deoxynivalenol (DON), greatly threatening food and feed safety (Buerstmayr et al., 2002; Bai and Shaner, 2004).

Developing and growing genetically resistant varieties is the most effective approach for managing the disease. Since about 1997, wheat breeding programs in Canada have been engaged in the search for FHB resistance and the development of new cultivars resistant to the disease. New varieties that contribute to a reduction in disease infection through improved resistance are critical to the control strategy in commercial production systems. In addition, improved kernel quality from reduced Fusarium damaged kernels (FDK) and low DON accumulation are critical to the successful marketing and end-use of the grain. Although there is much breeding effort underway and several adapted cultivars with improved resistance levels have been developed (DePauw et al., 2011; Cuthbert et al., 2017), to date, FHB resistance is not complete compared to resistance for other diseases such as leaf or stem rust because breeding for FHB resistance is complex.

Resistance to FHB is a quantitative trait with low to moderate heritability. The disease is governed by many minor effects of QTL that are subjected to strong QTL-by-environment interactions. FHB resistance was also reported to be associated with several agronomic and morphological traits, which makes the breeding effort more challenging. Various mapping studies investigated the association of FHB resistance with plant height and commonly reported negative correlations (Hilton et al., 1999; Buerstmayr et al., 2000; Somers et al., 2003; Srinivasachary et al., 2009; Lu et al., 2013). The co-incidence of QTL for FHB resistance and plant height was reported to have a genetic basis like tight linkage or pleiotropy rather than being the result of an escape mechanism (Schmolke et al., 2005; Draeger et al., 2007; Srinivasachary et al., 2008a; Srinivasachary et al., 2008b; Giancaspro et al., 2016). Days to heading were also reported to be negatively correlated with FHB resistance, with late heading being more resistant and attributed to a disease escape event (Emrich et al., 2008). Spike traits such as the presence and size of awns have been shown to be also associated with FHB resistance (Gervais et al., 2003; Tamburic-Ilincic et al., 2007; Srinivasachary et al., 2008a). Understanding the relationship between FHB resistance and these phenological and morphological traits is crucial for the development of resistant cultivars. Breeding programs can be seriously hampered if FHB resistance is linked to undesirable traits; therefore, simultaneous evaluation and selection are required while breeding for FHB resistance.

With the advent of next-generation sequencing technology, a high quantity of single-nucleotide polymorphism (SNP) markers have been developed and utilized to generate high-resolution genetic maps to locate QTL more precisely on known chromosome regions. The Illumina iSelect 90K wheat chip (Wang et al., 2014), which corresponds to a high-density SNP genotyping array with about 90,000 gene-associated SNPs, was developed as a powerful tool to characterize genetic variation in wheat and to provide a high-resolution dissection of complex traits such as FHB resistance. High-density linkage maps based on 90K iSelect markers are expected to improve the resolution of FHB resistance QTL analysis and enable the identification of markers desirable for marker-assisted selection (MAS) and backcrossing (Sari et al., 2018).

Various FHB resistance sources, mainly from Asia, Europe, and South America (Rudd et al., 2001), have been the focus of numerous genetic studies and have been used for QTL identification by most breeding programs worldwide (Buerstmayr et al., 2009). The Chinese spring wheat Sumai3 was widely used as a resistance source for its remarkably high resistance to fungal spread (Waldron et al., 1999; Anderson et al., 2001; Cuthbert et al., 2006). Resistance breeding efforts around the world depend heavily on Sumai3 and its derivatives, mainly as sources of the most commonly known *Fhb1* along with other genes such as *Fhb2* and *Fhb5* (Brar et al., 2019). Sumai3 derivatives have been used not only as donors of “active” resistance factors represented by FHB resistance loci but also as a source of “passive” resistance factors. For example, in the works by Lionetti et al. (2015) and Giancaspro et al. (2018), biochemical traits linked to cell-wall structure have been successfully transferred from a Sumai3-derived resistance line to susceptible durum cultivars, leading to the attainment of tolerant genotypes showing increased levels of FHB resistance measured as reduced severity, incidence, and lower accumulation of kernel DON levels. The moderately resistant cultivar Frontana from Brazil has been another widely studied wheat variety as a useful source of resistance to FHB disease (Singh et al., 1995; Steiner et al., 2004; Szabó-Hevér et al., 2012; Szabó-Hevér et al., 2014). Frontana showed the ability in *in vitro* experiments to degrade and tolerate high DON levels (Miller and Arnison, 1986; Wang and Miller, 1988). Szabó-Hevér et al. (2012) mapped QTL for resistance to FHB incidence in Frontana on chromosomes 2B, 3A, 4B, 5A, and 6B. In another study involving Frontana as a resistance source, Szabó-Hevér et al. (2014) identified QTL linked to FHB incidence, severity, FDK, and DON resistance on chromosomes 1B, 2D, 3B, 5A, 5B, and 6B. The QTL associated with DON accumulation alone was reported on chromosomes 3A, 4B, 7A, and 7B.

The Canadian spring wheat breeding programs have been making several crosses between adapted germplasm and sources of FHB resistance to transfer the resistance genes into the adapted background. The Canada Western Red Spring Wheat (CWRS) Carberry (DePauw et al., 2011) derives from the cross Alsen/Superb, where Alsen has Sumai3 in its pedigree. Superb has Frontana in its pedigree (CIERA: Yates et al., 2018). AC Cadillac, which derives from the cross BW90*3/BW553 (DePauw et al., 1998), also has Frontana in its background. Carberry shows moderate resistance, whereas AC Cadillac shows intermediate- to moderately susceptible reactions to FHB. In the present study, we used a doubled haploid population derived from the cross between an FHB moderately resistant cultivar, Carberry, and a moderately susceptible cultivar AC Cadillac to identify and localize QTL responsible for the expression of resistance to FHB and to investigate their association with plant height, maturity, days to heading, and awnedness.

## Materials and methods

### Plant material

A population of 811 doubled haploid (DH) lines was produced from a cross of Carberry/AC Cadillac using the maize pollen method described by Humphreys and Knox (2015). From the 811 lines, 261 were selected for height, maturity, straw strength, leaf and stem rust resistance similar to Carberry. The selected lines were evaluated for FHB incidence, severity, days to heading, days to maturity, awnedness, and plant height. Subsequently, the entire set of DH lines was evaluated to expand the population to 811 lines. Hereafter, the 261 set of lines will be referred to as the 261 set and the 811 set of lines as the 811 set.

### Disease assessment

FHB incidence and severity were assessed on 261 DH lines of the Carberry/AC Cadillac population, parents, and checks at an Agriculture and Agri-Food Canada (AAFC) FHB nursery near Portage la Prairie (PLP), Manitoba, in 2010, 2011, and 2013. AC Cadillac was not included in the 2010 PLP FHB nursery. Each DH line was planted in a single 1-m-long row using a six-row cassette Row XL, Wintersteiger planter. The rows were spaced 30 cm apart. Borders of 0.5 m were left between each set of six rows. The left row of each set of six rows was planted to either a control genotype or a rust disease spreader. The other five rows were planted with experimental lines.

The FHB inoculum used for the field inoculation to initiate disease at PLP originated from a mixture of four aggressive *F. graminearum* isolates M9-07-1 (3-ADON), M7-07-1 (3-ADON), M1-07-1 (15-ADON), and M3-07-2 (15-ADON) described by Gilbert and Woods (2006). The FHB corn-spawn inoculum was produced as described by Bokore et al. (2017). Briefly, sterile corn seed was inoculated in a laminar flow hood with these four isolate cultures. Following the incubation period, the corn kernel inoculum was dried. The dried corn was then packed in mesh bags and stored in a cold room at about 0 °C for its use in the experiment. These *F. graminearum* isolate-colonized corn grains were broadcasted between the rows at a rate of 20 g/m^2^ at the end of the tillering stage, which was approximately 2 to 3 weeks prior to flowering (Zadoks stage 58). To promote FHB symptom development, an overhead low-pressure mist irrigation system was programmed to apply about 12 mm of water three times a week to maintain a humid environment in the plots. FHB symptoms were scored between 22 and 25 days after the majority of the lines had completed anthesis, and a differential response to FHB was expressed in the controls.

The 811 DH lines of the Carberry/AC Cadillac population, along with parents and checks, were evaluated for FHB incidence and severity at AAFC’s FHB nurseries near Morden (MDN), MB, in 2015, 2016, and 2017, and Brandon (BDN), MB, in 2016 and 2017. At both locations, the lines were planted in a single 1-m-long row using a six-row cassette Row XL, Wintersteiger planter. The rows were spaced 30 cm apart, with borders of 0.5 m between each set of six rows. The DH lines in the experiments were unreplicated, while the parents and checks, including Sumai3, FHB37, and CDC Teal, were repeated ten times. *F. graminearum* corn kernel inoculum was prepared by modifying the protocol of Gilbert and Woods (2006). The inoculum was prepared using steam table pans (10 cm) using four *F. graminearum* isolates (M9-07-1 (3-ADON), M7-07-1 (3-ADON), M1-07-1 (15-ADON), and M3-07-2 (15-ADON). Each isolate was inoculated in individual pans containing sterile corn and incubated for 1 month. After the corn dried, it was stored in plastic tubs at 4°C until use. At Morden, the inoculum was dispersed at a rate of 8 g per row, two times at weekly intervals, starting when the earliest lines were at the four- to five-leaf stage. The inoculum application was followed by irrigation three times a week (Monday, Wednesday, and Friday) using Cadman Irrigation Travelers with Briggs booms. At BDN, the inoculum was also broadcasted twice. The first application was 6 weeks after planting, followed by another application 2 weeks after the first application. Overhead irrigation was applied three times a week after the inoculation to promote the development of the disease and the spread of *F. graminearum* spores from the inoculated corn.

At 21 days post-anthesis, the two components of resistance, FHB incidence (initial infection) and FHB severity (fungal spread), were visually scored using a scale of 0% to 100% (Stack and McMullen, 1998). FHB incidence was the percentage of infected spikes over total spikes per row, while FHB severity was the averaged estimated percentage of infected spikelets per head for all spikes in the row. FHB index was calculated using the following formula: FHB index = (percent disease incidence × percent disease severity)/100.

### Agronomic and morphological assessment

Plant height (PH), days to heading (DTH), and days to maturity (MAT) were recorded in an irrigated seed increase nursery near Swift Current, SK, on the 261 and 811 sets of the population, parental lines, and checks. Plant height was measured in 2010 and 2011 (two locations) on the 261 set, and in 2012, 2013 (two locations), and 2014 on the 811 lines from the soil surface to the tip of spikes, excluding the awns. Similarly, DTH was recorded in 2011 (two locations) on the 261 set and in 2013 (two locations) and 2014 on the 811 set when 50% of the heads emerged from the boot. Days to maturity were recorded in 2011 on the 261 set and in 2012, 2013 (two locations), and 2014 on the 811 set when 80% or more of the plots had yellow heads and achieved physiological maturity (30% to 35% moisture on a wet weight basis). The awn phenotype in the 811 set was scored as Carberry type for the presence of long awns (score = 1) or AC Cadillac type for tipped awns (score = 0) in an irrigated increase nursery near Swift Current during the 2012 growing season.

### Molecular genotyping and high-density genetic map construction

The procedure for the DNA extraction and PCR analysis on the 261 set of lines was previously described by Singh et al. (2016). Briefly, the genomic DNA of the lines and parents used for simple sequence repeat (SSR) marker genotyping was extracted from 3-cm segments of primary leaves using the wheat and barley DNA extraction in 96-well plate protocol (http://maswheat.ucdavis.edu/PDF/DNA0003.pdf) with some modifications. For Diversity Array Technology (DArT) analysis, genomic DNA of the lines and parents was extracted according to a protocol published by Triticarte (http://www.triticarte.com.au/pdf/DArT_DNA_isolation.pdf) with some modifications as described by Singh et al. (2013). The DArT® marker analysis was done by Triticarte Pty. Ltd. Yarralumla, ACT, Australia (www.triticarte.com.au).

A total of 58 SSR and 578 DArT markers were polymorphic between the two parents and used in generating a genetic linkage map of the 261 set of lines. The JoinMap® 4.0 software was used to develop the linkage map using the regression mapping option and groupings (Van Ooijen, 2006). Centimorgan (cM) values were calculated according to the Haldane mapping function. The validity of the linkage groups was confirmed with known chromosomal locations of markers determined through the GrainGenes website (http://wheat.pw.usda.gov/GG2/index.shtml). Additionally, in order to cross-reference markers associated with resistance QTL identified from the two sets using the DArT/SSR map and the high-density SNP map, we generated a genetic map incorporating together the SNP, DArT, and SSR markers.

For the 811 set, the genomic DNA of the parents and DH lines was extracted from young leaves with the DNeasy 96 Plant DNA Extraction Kit (QIAGEN Science, MD, USA). Genotyping was done with the 90K iSelect SNP Genotyping Assay (Illumina Inc., San Diego, CA, USA). Genotypes were called with the automatic clustering algorithm in Genome Studio V2011.1, followed by manual adjustment to recover polymorphic markers that were not correctly called by the algorithm. In addition to SNP markers, selected microsatellite markers *Xbarc147*, *Xbarc75*, *Xgwm389*, *Xgwm533*, and *Umn10* (Cuthbert et al., 2006; Liu et al., 2008; Buerstmayr et al., 2009) previously associated with or in the genomic region of the FHB gene *Fhb1*, as well as an STS marker *Xsts3B-142* near *Fhb1* (Cuthbert et al., 2006), were also used to genotype the population. From the 811 set, only 775 lines were used to generate the genetic map using JoinMap®5.

### QTL analysis

QTL analysis was performed with IciMapping Version 4 (Meng et al., 2015) using the 261-set DArT/SSR map and FHB data from PLP. The IciMapping software option Inclusive Composite Interval Mapping with additive effect (ICIM-ADD) was used to identify molecular markers significantly associated with FHB resistance and agronomic traits. ICIM is based on the stepwise regression of simultaneous consideration of all marker information (Li et al., 2007; Li et al., 2008). A logarithm of odds (LOD) threshold of 2.5 cM was specified from a 1,000 permutation test to declare significant QTL. Additionally, using the DArT/SSR map and a high-density SNP map on the FHB data of the 261 set from PLP, MDN, and BDN, a multiple QTL mapping (MQM) of the MapQTL software was used to determine QTL. The aim of the analysis was to cross-reference markers associated with FHB resistance QTL identified with a DArT/SSR map and SNP markers. Furthermore, to investigate the effect of the 4B *Rht-B1* reduced height gene on the FHB reaction, we pooled 336 lines that carried molecular variant for Carberry *Rht-B1* type on chromosome 4B, and we performed QTL analysis using FHB data collected from MDN in 2015, 2016, and 2017 and BDN in 2016 and 2017.

For the 775 DH set, QTL analysis was performed using the MapQTL6^®^ software (Van Ooijen, 2009). A nonparametric Kruskal–Wallis (KW) test was employed to detect an association between markers and traits individually. Interval mapping (IM) analysis was then performed to select markers significantly associated with the trait to constitute an initial set of co-factors. A backward elimination procedure was applied to the initial set of co-factors. Automatic co-factor marker detection declaring significant markers at *p* < 0.02 as well as manual co-factor selection were performed, and the selected co-factor markers were used in MQM (Jansen and Stam, 1994). Genome-wide thresholds of the LOD scores for significant (*p* < 0.05) QTL were determined by 1,000 permutations within MapQTL^®^, and the significance of the detected QTL was confirmed. The Basic Local Alignment Search Tool (BLAST) against the genome sequence of Chinese Spring wheat on the IWGSC RefSeq v1.0 was used to compare the physical positions of selected QTL.

### Statistical analysis

To determine the broad-sense heritability (*H*) of the FHB traits, analysis of variance (ANOVA) was performed using a mixed model approach in the Statistical Analysis System (SAS) software version 9.3 (SAS Institute Inc., Cary, NC, USA). During the analysis, the DH lines were considered a fixed variable, whereas test locations and years were considered random variables. The broad sense heritability was calculated from the variance components. Spearman’s correlation coefficients between FHB traits recorded at the different locations were estimated using PROC CORR in SAS. We also estimated the correlation coefficients between FHB traits obtained from the different locations and the adjusted mean value from multiple locations of plant height, days to heading, and maturity.

## Results

### Trait variation in the 261 set of lines

**Figure 1:**
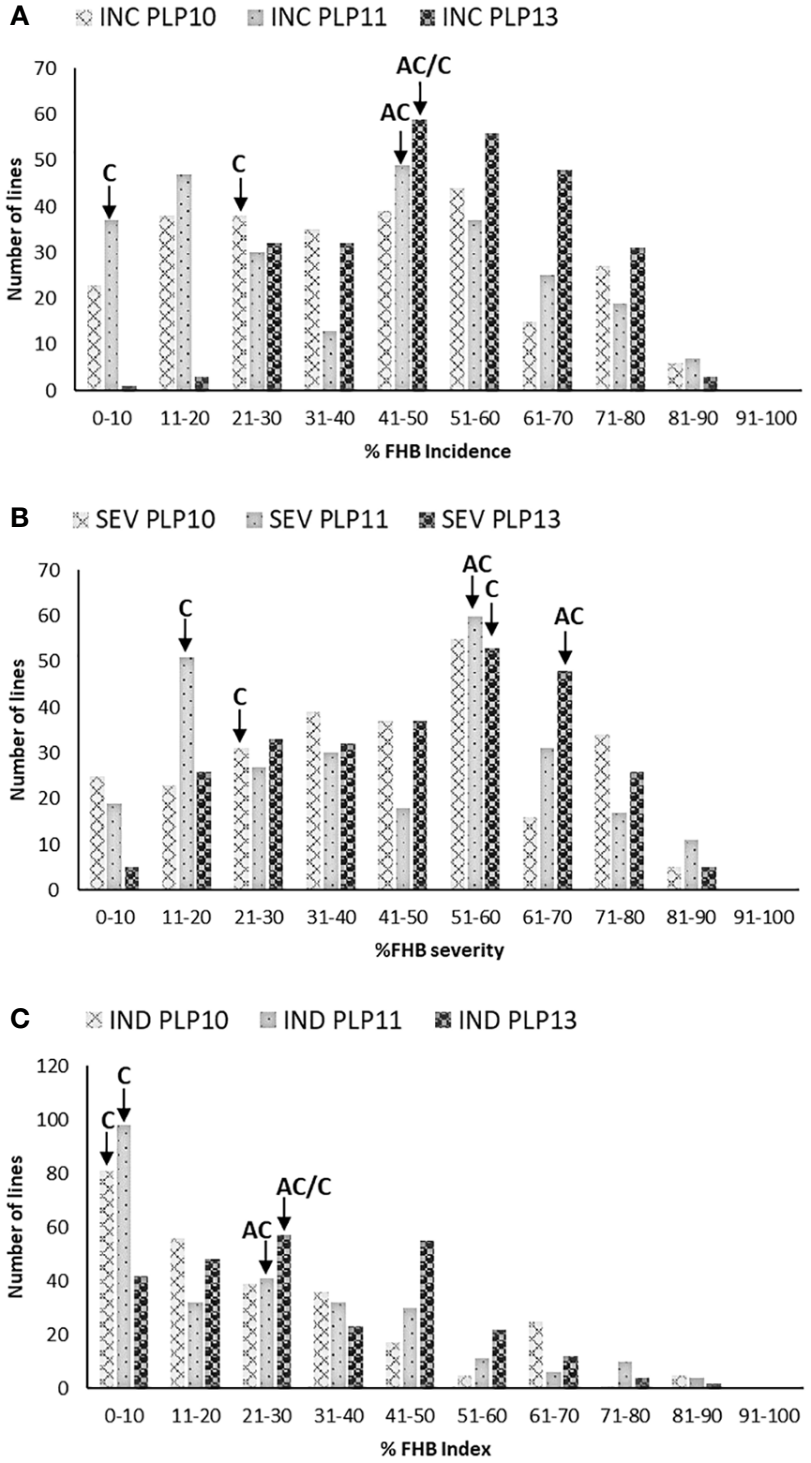
Frequency distribution of 261 Carberry (C)/AC Cadillac (AC) DH lines grown at Portage La Prairie (PLP) in 2010, 2011, and 2013 for **(A)** FHB incidence (INC), **(B)** FHB severity (SEV), and **(C)** FHB index (IND). The placement of the parents along the distribution is denoted by arrows. No data were available for AC Cadillac in 2010.

**Figure 1** presents the frequency distribution of FHB traits (A) incidence, (B) severity, and (C) index of PLP. Similar FHB pressures were observed in 2010 and 2011, whereas higher disease symptoms were observed in 2013 than in 2010 and 2011. There was highly significant variation among the lines for all the FHB traits (**Table 1**). The frequency distributions of the different FHB parameters were continuous in all three environments. Transgressive segregates, more resistant or susceptible than parents, were also observed. The presence of transgressive segregant lines indicates that both parental lines have additively contributed to the resistance. AC Cadillac and Carberry displayed different reactions to FHB infection, with the difference between the means of their FHB traits being significant at PLP in 2011 but not in 2013. The lack of significance in 2013 could be associated with the interaction between the disease and the environment. Overall, the more resistant parent, Carberry, was less infected than the moderately susceptible parent, AC Cadillac. A comparison between the two parents was not possible at PLP in 2010, as Carberry was missing from the trial.

**Table 1:** Analysis of variance of line means, heritability (*H*), means of parents and population, minimum and maximum values of Fusarium head blight, plant height, and days to heading traits for the 261 sets of lines of the Carberry/AC Cadillac population.

Traits	*p*-value^a^	*H* ^b^	Environment	Carberry µ, AC Cadillac µ	Population			
					Mean	Min	Max	Std error
FHB incidence	^***^	0.60	PLP 2010		44.6	5	90	1.4
			PLP 2011	10.8, 45^**^	41.8	2	90	1.5
			PLP 2013	50, 45 (ns)	55.6	10	90	1.0
FHB severity	^***^	0.62	PLP 2010		47.6	5	90	1.4
			PLP 2011	12.5, 53.3^***^	45.8	5	90	1.5
			PLP 2013	55, 65 (ns)	51.8	10	90	1.2
FHB index	^***^	0.64	PLP 2010		25.5	0.25	81	1.3
			PLP 2011	1.5, 26^**^	24.6	0.1	81	1.3
			PLP 2013	27.5, 29 (ns)	31.4	1	81	1.1
Plant height	^***^	0.89	SC 2010	69.5, 89^*^	88.6	72	110	0.5
			L1-SC 2011	78, 99.6^***^	83.3	68	102	0.4
			L2-SC 2011	79.7, 101.5^***^	87.4	71	108	0.5
Days to heading	^***^	0.73	L1-SC 2011	50.4, 53.1^***^	51.3	46	58	0.1
			L2-SC 2011	56.2, 57.7^**^	56.6	52	61	0.1

SC, Swift Current; L1-SC, location 1 near Swift Current; L2-SC, location 2 near Swift Current; PLP, Portage La Prairie.

^*^p ≤ 0.05, ^**^p ≤ 0.01, and ^***^p ≤ 0.001—significance of difference between means of parents based on t-test.

a p-values of line variance.

b Broad sense heritability. ns, non-significant.

There were statistically significant variations among the 261 set of lines for plant height, days to heading, and maturity (**Table 1**). Plant height ranged from 72 to 102 cm at Swift Current in 2010, 68 to 102 cm at Swift Current location 1 in 2011, and from 71 to 108 cm at location 2 in 2011. Across the years, the mean plant height of Carberry was 21.2 cm shorter than AC Cadillac and headed out 2.1 days earlier than AC Cadillac. Days to heading ranged from 46 to 58 days at location 1 in 2011 and 52 to 61 days at location 2 in 2011. The maturity date of the population ranged from 95 to 100 days in 2011. On average, AC Cadillac reached maturity 1.5 days earlier than Carberry. There were significant statistical differences between the two parents for plant height, days to heading, and maturity in 2010 and 2011 in all the locations.

### Trait variation on the 811 set of lines

**Figure 2:**
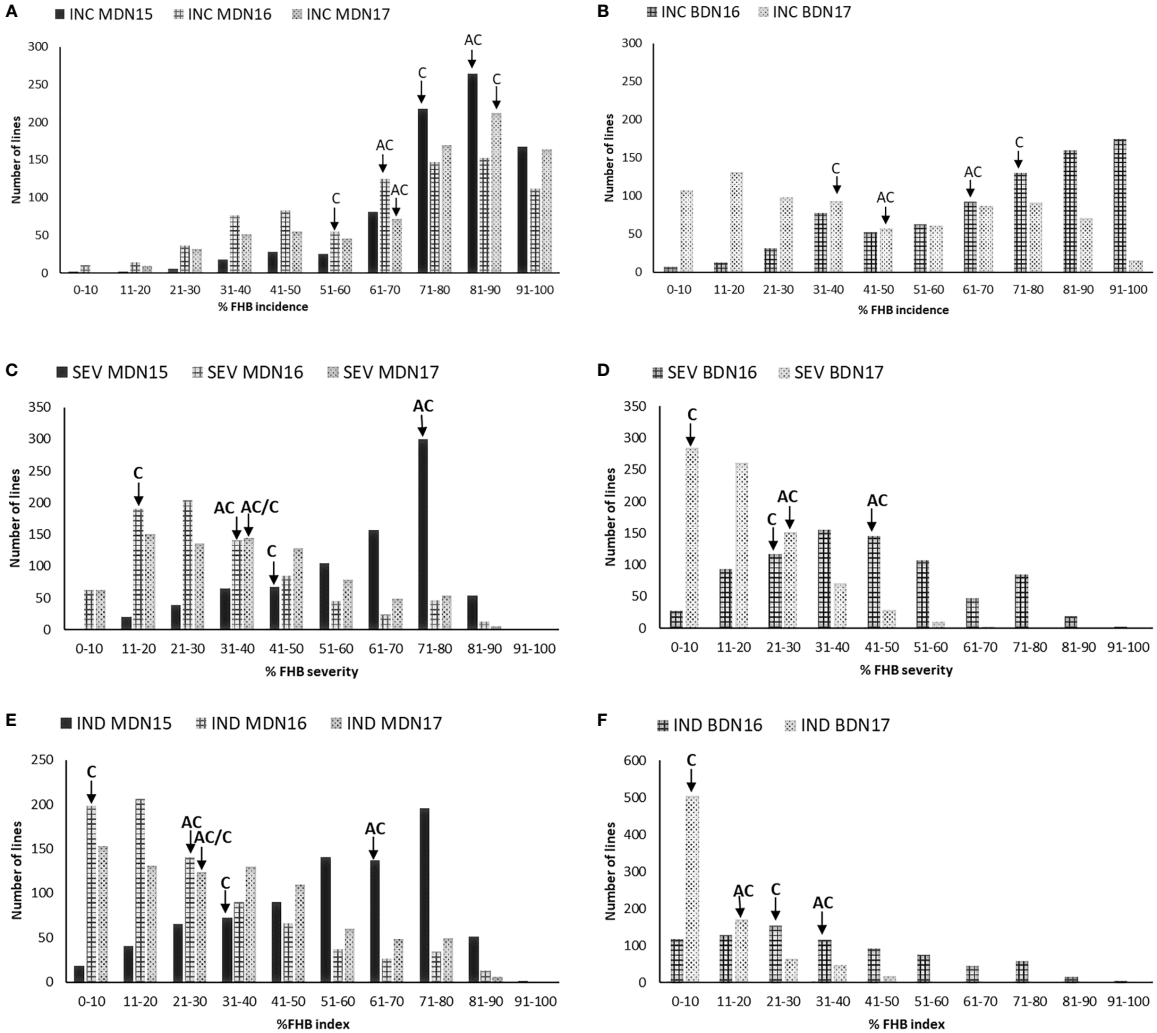
Frequency distribution of 811 Carberry (C)/AC Cadillac (AC) DH lines grown at Morden (MDN) in 2015, 2016, and 2017 and Brandon (BDN) in 2016 and 2017 for FHB incidence (INC) in **(A, B)**, severity (SEV) in **(C, D)**, and FHB index (IND) in **(E, F)**. The placement of the parents along the distribution is denoted by arrows.

Lines with higher FHB incidence, severity, and index than both parents were observed across environments. Overall, the highest disease development was observed in the MDN 2015 environment, whereas the lowest disease development was observed at BDN in 2017 (**Table 2**). Significant differences were observed between AC Cadillac and Carberry for the FHB incidence at MDN in 2015, the FHB severity at MDN in 2016 and 2017 and BDN in 2017, and for the FHB index at MDN in 2015 and 2016 (**Table 2**). Significant variation was also observed among the population lines for all the FHB traits. The frequency distribution of the 811 DH lines was continuous for all the FHB traits in all the environments (**Figures 2A–F**). The population was skewed for FHB incidence at MDN, with a preponderance of lines in the susceptible end of the distribution, as demonstrated in **Figure 2A**. At BDN, a preponderance of susceptible lines was observed in 2016 and of resistant lines in 2017. For the MDN 2016 and 2017 and BDN 2017 FHB severity, the distribution of the population was skewed with a preponderance of resistant lines, whereas at MDN in 2015, the population was skewed toward susceptibility. However, the distribution in the BDN 2016 environment was close to normal. Except for MDN 2015, the population distribution for the FHB index was skewed toward the resistant end, as shown in **Figures 2D, E**.

**Table 2:** Analysis of variance of line means, heritability (*H*), means of parents and population, minimum and maximum values of Fusarium head blight, plant height, days to heading, and maturity traits for the 811 Carberry × AC Cadillac DH lines.

Traits	*p*-value^a^	*H* ^b^	Environment	Carberry µ, AC Cadillac µ	Population			
					Mean	Min	Max	Std error
FHB incidence	^***^	0.53	MDN 2015	77.5, 90.5^***^	82.4	4	100	0.5
			MDN 2016	58.5, 69 (ns)	69.0	10	100	0.8
			BDN 2016	79.5, 61.5^*^	73.0	10	100	0.8
			MDN 2017	84.5, 67^*^	77.0	10	100	7
			BDN 2017	34, 41.5 (ns)	46.0	1	100	1
FHB severity	^***^	0.61	MDN 2015	42, 76^***^	65.0	0	95	0.6
			MDN 2016	17.5, 39^***^	34.0	5	90	0.7
			BDN 2016	33, 47^*^	44.0	10	100	0.7
			MDN 2017	32, 34.6^*^	38.0	5	90	0.7
			BDN 2017	9.5, 23.5^***^	20.0	5	75	0.4
FHB index	^***^	0.49	MDN 2015	32.7, 68.9^***^	55.6	0	95	0.7
			MDN 2016	10.7, 28^***^	26.6	0.5	90	0.7
			BDN 2016	25.9, 31.7 (ns)	35.4	1	100	0.8
			MDN 2017	27.3, 24.6 (ns)	32.5	1	90	0.8
			BDN 2017	3.3, 10.8^**^	11.0	0.1	71.3	0.4
Plant height	^***^	0.78	SC 2012	82.5, 102.3^***^	92.2	66	120	0.3
			L1-SC 2013	92.9, 120.71^***^	104.9	74	138	0.5
			L2-SC 2013	89.8, 112.82^***^	101.1	64	132	0.4
			SC 2014	91.2, 112^***^	100.3	66	134	0.4
Days to heading	^***^	0.90	L1-SC 2013	54.6, 55.5^**^	55.5	51	66	0.1
			L2-SC 2013	54.1, 55.6^***^	54.6	49	63	0.1
			SC 2014	59.2, 61.2^***^	60.4	57	71	0.1
Maturity	^***^	0.62	SC 2012	94.4, 94.3 (ns)	94.0	77	103	0.1
			L1-SC 2013	101.9, 98.4^***^	99.7	93	106	0.1
			L2-SC 2013	102.9, 101.5^***^	101.7	96	107	0.1
			SC 2014	100.8, 100.2^***^	100.3	80	103	0.1

^*^p ≤ 0.05, ^**^p ≤ 0.01, and ^***^p ≤ 0.001—significance of difference between means of parents based on t-test.

a p-values of line variance.

b Broad sense heritability. ns, non-significant.

The plant height of the population ranged from 66 to 120 cm at Swift Current in 2012, 74 to 138 cm in 2013 at Swift Current location 1, 64 to 132 cm in 2013 at Swift Current location 2, and 66 to 134 cm at Swift Current in 2014. Days to heading ranged from 51 to 66 days at location 1 in 2013, 49 to 63 days at location 2 in 2013, and 57 to 71 days at Swift Current in 2014. The maturity date ranged from 77 to 103 days in 2012, 93 to 103 days at location 1 in 2013, 96 to 107 days at location 2 in 2013, and 80 to 103 days at Swift Current in 2014. Over all the years, Carberry was 22.8 cm shorter than AC Cadillac, and it headed 1.5 days earlier than AC Cadillac.

### Trait correlations

The results of the correlation analysis are presented in **Table 3** for the 261 set and **Table 4** for the 811. Overall, associations among FHB traits averaged over locations were weak to moderate but were significant. In both sets, the FHB traits showed negative and highly significant weak to moderate correlations with PH, such that shorter plants tended to show higher FHB symptoms. Negative and weak correlations were also observed between the FHB traits and DTH for the 811 set, whereas the 261 set showed a negative association only in 2013, but otherwise were positively correlated with FHB traits in 2010 and 2011 or the correlation was not significant. The correlation of FHB traits with maturity showed significance only in 2013 and was negative and low for the 261 set. Negative and highly significant low-to-moderate associations between FHB traits and maturity were revealed for the 811 set.

**Table 3:** Spearman’s correlation coefficients between Fusarium head blight traits measured over environments, plant height, days to heading, and maturity on the 261 Carberry × AC Cadillac DH lines.

Trait	Environment	FHB Incidence		
		PLP 2010	PLP 2011	PLP 2013
FHB Incidence	PLP 2010	-	0.48***	0.34***
	PLP 2011	-	-	0.20**
Plant height	3 ENV	-0.47***	-0.22***	-0.23***
Days To heading	2 ENV	ns	0.24***	-0.16**
Maturity	SC 2011	ns	ns	-0.15*
		FHB Severity		
		PLP 2010	PLP 2011	PLP 2013
FHB Severity	PLP 2010	-	0.48***	0.34***
	PLP 2011	-	-	0.25***
Plant height	3 ENV	-0.51***	-0.18**	ns
Days To heading	2 ENV	0.13*	0.22***	ns
Maturity	SC 2011	ns	ns	-0.13*
		FHB Index		
		PLP 2010	PLP 2011	PLP 2013
FHB Index	PLP 2010	-	0.52***	0.35***
	PLP 2011	-	-	0.26***
Plant height	3 ENV	-0.49***	-0.22***	-0.15*
Days To heading	2 ENV	ns	0.21***	-0.14*
Maturity	SC 2011	ns	ns	-0.15*

PLP, Portage La Prairie; SC, Swift Current; 2 ENV, trait mean value of two environments; 3 ENV, trait mean value of three environments.

ns, nonsignificant for p > 0.05; ^*^p ≤ 0.05; ^**^p ≤ 0.01; ^***^P ≤ 0.001.

**Table 4:** Spearman’s correlation coefficients between Fusarium head blight traits measured over environments, plant height, days to heading, and maturity on the 811 Carberry × AC Cadillac DH lines.

Trait	Environment	FHB Incidence				
		MDN 2015	MDN 2016	BDN2016	MDN 2017	BDN 2017
FHB Incidence	MDN 2015	-	0.32***	0.22***	0.39***	0.35***
	MDN 2016	-	-	0.26***	0.44***	0.43***
	BDN2016	-	-	-	0.36***	0.34***
	MDN 2017	-	-	-	-	0.53***
Plant height	4 ENV	-0.25***	-0.36***	-0.28***	-0.50***	-0.38***
Days To heading	3 ENV	-0.13***	-0.15***	-0.42***	-0.24***	-0.15***
Maturity	4 ENV	-0.20***	-0.28***	-0.23***	-0.42***	-0.32***
		FHB Severity				
		MDN 2015	MDN 2016	BDN2016	MDN 2017	BDN 2017
FHB Severity	MDN 2015	-	0.37***	0.40***	0.38***	0.45***
	MDN 2016	-	-	0.41***	0.52***	0.51***
	BDN2016	-	-	-	0.45***	0.48***
	MDN 2017	-	-	-	-	0.54***
Plant height	4 ENV	-0.09**	-0.26***	-0.22***	-0.42***	-0.10**
Days To heading	3 ENV	-0.10**	ns	-0.30***	-0.11**	ns
Maturity	4 ENV	ns	-0.20***	-0.17***	-0.37***	ns
		FHB Index				
		MDN 2015	MDN 2016	BDN2016	MDN 2017	BDN 2017
FHB Index	MDN 2015	-	0.38***	0.37***	0.41***	0.46***
	MDN 2016		-	0.40***	0.52***	0.54***
	BDN2016			-	0.46***	0.53***
	MDN 2017				-	0.60***
	BDN 2017					-
Plant height	4 ENV	-0.16***	-0.29***	-0.27***	-0.45***	-0.24***
Days To heading	3 ENV	-0.13***	ns	-0.35***	-0.16***	-0.14***
Maturity	4 ENV	-0.12**	-0.24***	-0.23***	-0.40***	-0.20***

MDN, Morden; BDN, Brandon; 3 ENV, trait mean value of three environments; 4 ENV, trait mean value of four environments.

ns, nonsignificant for p > 0.05; ^*^p ≤ 0.05; ^**^p ≤ 0.01; ^***^p ≤ 0.001.

### Linkage map construction

For the 261 set, the linkage map with 21 linkage groups was constructed using 634 polymorphic DArT and SSR markers. The linkage groups formed were anchored to the 21 wheat chromosomes for an approximate map length of 2,101.6 cM. For the expanded population, 775 lines were used to generate the genetic map with 29 linkage groups using a total of 6,806 SNP markers, and the total genetic distance covered by the linkage map was 4,527.5 cM. An alternative to this high-density SNP map was developed using a combination of 7,370 DArT, SSR, and SNP markers and 775 DH set, covering a total map length of 6,488.4 cM in the wheat genome.

### FHB QTL identified in the 261 set of lines

Using the DArT/SSR genetic map, ICIM QTL analysis on the 261 DH lines evaluated at PLP identified four QTL associated with FHB resistance on chromosomes 2A (designated as *Qfhb.spa-2A*), 3B (*Qfhb.spa-3B.1*), 4B (*Qfhb.spa-4B*), and 5A (*Qfhb.spa-5A.1*) (**Table 5A**). Carberry contributed resistance alleles at *Qfhb.spa-2A, Qfhb.spa-3B.1*, and *Qfhb.spa-5A.1*, whereas AC Cadillac contributed the resistance allele for *Qfhb.spa-4B*. The QTL *Qfhb.spa-3B.1*, *Qfhb.spa-4B*, and *Qfhb.spa-5A.1* were associated with both FHB incidence and severity in two out of three environments, but *Qfhb.spa-2A* was associated only with FHB incidence in the PLP 2013 environment. The significant QTL LOD scores ranged from 4.1 to 14.1; the highest LOD score was associated with the *Qfhb.spa-4B* for FHB severity at PLP in 2010. *Qfhb.spa-4B* also explained the greatest amount of phenotypic variation associated with the same FHB trait. *Qfhb.spa-3B.1* was effective against FHB incidence in 2010 and FHB incidence and severity in 2013. *Qfhb.spa-4B* and *Qfhb.spa-5A.1* were expressed in 2010 and 2011 by reducing the FHB incidence and severity, but both did not show significant expression in 2013.

**Table 5:** Summary of Fusarium head blight (FHB) QTL detected from the 261 sets of the Carberry × AC Cadillac population using DArT/SSR map and SNP map, the number of test environments, phenological and morphological traits associated with FHB QTL, markers associated with the highest LOD values, and FHB resistance allele contributing parent of evaluated at PLP in 2010, 2011, and 2013; Morden (MDN) in 2015, 2016, and 2017; and Brandon (BDN) in 2016 and 2017.

Chr.	QTL	Total number of environments analysed	Number of environments where QTL identified	Location / year	FHB trait	Co-location with	Markers with the highest LOD	Highest LOD	Highest PVE (%)	Source of FHB resistance
A) QTL analysis by ICIM for the 261 DH set using DArT/SSR map at PLP in 2010, 2011, and 2013										
2A	*Qfhb.spa-2A*	3	1	PLP13	INC		*wPt-9320/wPt-665330*	4.1	7.6	Carberry
3B	*Qfhb.spa-3B.1*	3	2	PLP10,13	INC,SEV		*wPt-744251/wPt-2757*	11.2	18.5	Carberry
4B	*Qfhb.spa-4B*	3	2	PLP10,11	INC,SEV	MAT, PH	*wPt-744434/Xwmc617*	14.1	23.4	AC Cadillac
5A	*Qfhb.spa-5A.1*	3	2	PLP10,11	INC,SEV		*Xgwm595/wPt-1038*	10.8	16.3	Carberry
B) QTL analysis by MQM for the 261 DH set using DArT/SSR map at Morden in 2015, 2016, 2017 and Brandon in 2016 and 2017										
2A	*Qfhb.spa-2A*	5	1	MDN17	SEV IND		*wPt-0873*	3.5	5.1	Carberry
3A	*Qfhb.spa-3A.1*	5	1	MDN15	INC SEV IND		*rPt-9057/rPt-2940*	3.9	5.9	AC Cadillac
3B	*Qfhb.spa-3B.1*	5	5	MDN15,16,17; BDN16,17	INC SEV IND		*wPt-744251*	11.3	16.9	Carberry
4B	*Qfhb.spa-4B*	5	3	MDN16,17; BDN17	INC SEV IND	MAT, PH, DTH	*wPt-744434/Xwmc617*	7.2	11.2	AC Cadillac
5A	*Qfhb.spa-5A.1*	5	3	MDN15,17; BDN17	INC SEV IND		*Xgwm595/wPt-1038*	8	11.6	Carberry
C) QTL analysis by MQM for the 261 DH set using SNP map at PLP in 2010, 2011, and 2013										
2A	*Qfhb.spa-2A*	3	1	PLP13	INC IND		*BS00041816_51/BS00022241_51*	3.5	5.8	Carberry
3B	*Qfhb.spa-3B.1*	3	1	PLP13	INC SEV IND		*BobWhite_c2453_340 /BobWhite_c2453_282*	11.7	20.2	Carberry
	*Qfhb.spa-3B.2*	3	1	PLP10	INC SEV IND		*Barc75/RAC875_c101155_64*	3.8	7	Carberry
4B	*Qfhb.spa-4B*	3	2	PLP10,11	INC SEV IND	PH, DTH	*BS00021984_51/wsnp_BF482960B_Ta_1_4*	10.3	17.9	AC Cadillac
5A	*Qfhb.spa-5A.1*	3	3	PLP10,11,13	INC SEV IND		*BobWhite_c8266_227*	23.8	33.8	Carberry
D) QTL analysis by MQM for the 261 DH set using SNP map at Morden in 2015, 2016, 2017 and Brandon in 2016 and 2017										
3A	*Qfhb.spa-3A.1*	5	1	MDN15	INC SEV IND	MAT	*wsnp_Ex_c10272_16842803*	6.5	8.2	AC Cadillac
3B	*Qfhb.spa-3B.1*	5	5	MDN15,16,17; BDN16,17	INC SEV IND		*BobWhite_c2453_340 /BobWhite_c2453_282*	11.7	20.2	Carberry
4B	*Qfhb.spa-4B*	5	3	MDN16,17; BDN17	INC SEV IND	PH, DTH	*BS00021984_51/wsnp_BF482960B_Ta_1_4*	9.6	16.9	AC Cadillac
5A	*Qfhb.spa-5A.1*	5	5	MDN15,16,17; BDN16,17	INC SEV IND		*BobWhite_c8266_227/BS00022864_51*	15	20.7	Carberry
	*Qfhb.spa-5A.2*	5	1	MDN17	INC	DTH	*Kukri_c86812_193/Excalibur_c60150_107*	6.5	9.2	AC Cadillac

INC, FHB incidence; SEV, severity; IND, index; PVE, phenotypic variation explained; MAT, maturity; PH, plant height; abbreviations for test locations followed by the last two digits of experiment years.

The second QTL analysis was an MQM analysis using the DArT/SSR genetic map and FHB data of the 261 DH set evaluated at MDN and BDN, which similarly detected *Qfhb.spa-2A*, *Qfhb.spa-3B.1*, *Qfhb.spa-4B*, and *Qfhb.spa-5A.1*, with an additional QTL detected on chromosome 3A (*Qfhb.spa-3A.1*) (**Table 5B**). The *Qfhb.spa-3B.1*, *Qfhb.spa-4B*, and *Qfhb.spa-5A.1* were detected in multiple environments, whereas the QTL on *Qfhb.spa-2A* and *Qfhb.spa-3A.1* were detected in single environments. Contributed by AC Cadillac, *Qfhb.spa-3A.1* explained a phenotypic variation of up to 5.9% in the FHB traits, which was detected in the MDN 2015 environment. The third QTL analysis, which used the high-density SNP map and the MQM option of the MapQTL on the 261 DH set and FHB data of PLP, revealed genomic regions that were associated with FHB resistance on chromosomes 2A (*Qfhb.spa-2A*), 3B (two loci, *Qfhb.spa-3B.1* and *Qfhb.spa-3B.2*), 4B (*Qfhb.spa-4B*), and 5A (*Qfhb.spa-5A.1*), similar to the other genetic maps (**Table 5C**). The analysis by MQM using the SNP map on the 261 set for the MDN and BDN environments revealed *Qfhb.spa-3A.1*, *Qfhb.spa-3B.1*, *Qfhb.spa-4B*, and *Qfhb.spa-5A.1*, with an additional QTL on chromosome 5A, *Qfhb.spa-5A.2* (**Table 5D**). The resistance alleles at *Qfhb.spa-2A*, *Qfhb.spa-3B.1*, *Qfhb.spa-3B.2*, and *Qfhb.spa-5A.1* were derived from Carberry, whereas the resistance alleles at *Qfhb.spa-3A.1*, *Qfhb.spa-4B*, and *Qfhb.spa-5A.2* were derived from AC Cadillac.

The *Qfhb.spa-3B.1* was revealed in all the MDN and BDN environments and in two out of three PLP environments. *Qfhb.spa-3B.1* was more consistently expressed than *Qfhb.spa-3B.2*, which was detected only in the PLP 2010 environment (**Table 5C**). *Qfhb.spa-3B.1* explains 8.7% to 11.7% of the variation in FHB incidence, 11.7% to 20.2% in FHB severity, and 8.6% to 19.5% in the FHB index, with the explained variation in the FHB trait reaching 20.2%. The *Qfhb.spa-3B.2* gave the highest explained phenotypic variation in the FHB severity of 7%. The *Qfhb.spa-5A.1* was expressed fairly consistently among the environments and accounted for the highest PVE in FHB incidence of up to 33.8% at PLP in 2011. The *Qfhb.spa-5A.2* explained a phenotypic variation of 9.2% in the FHB incidence. The other consistent QTL, *Qfhb.spa-4B*, explained up to 17.9% of the phenotypic variation that was observed at PLP in 2010. The QTL *Qfhb.spa-2A* and *Qfhb.spa-3A.1* were environment-sensitive and had a minor effect on reducing the FHB disease. Both QTL were detected in only one environment. Whereas *Qfhb.spa-2A* was associated with FHB incidence and index at PLP in the 2013 environment, *Qfhb.spa-3A.1* was associated with resistance to FHB incidence, severity, and index at MDN in 2015.

QTL analysis using the SNP/DArT/SSR integrated map on the 261 set identified nine genomic regions responsible for FHB resistance on chromosomes 2A (*Qfhb.spa-2A*), 3A (*Qfhb.spa-3A.1*, *Qfhb.spa-3A.2*), 3B (*Qfhb.spa-3B.1*, *Qfhb.spa-3B.2*), 4B (*Qfhb.spa-4B*), 5A (*Qfhb.spa-5A.1*), 6A (*Qfhb.spa-6A*), and 6D (*Qfhb.spa-6D*) (**Table 6**). Resistance alleles on the chromosomes 2A, 3A, 3B, 5A, and 6D QTL were contributed by Carberry, while those on the 3A, 4B, and 6A QTL were contributed by AC Cadillac. QTL on chromosomes 4B and 5A were effective in five test environments and explained the highest phenotypic variation of 34.4% in PLP 2011 for the QTL on 5A to reduce FHB incidence and 19.2% in PLP 2010 for the QTL on 4B to reduce FHB severity. QTL on chromosomes 2A, 6A, and 6D were only expressed in one environment: PLP 2013 for the 2A QTL, MDN 2017 for the 6A QTL, and PLP 2010 for the 6D QTL. Chromosome 3B carried two FHB resistance loci: a major-effect locus *Qfhb.spa-3B.1* observed in six environments and explaining the highest PVE of 20.9% to reduce FHB severity in MDN 2016, and a minor-effect locus *Qfhb.spa-3B.*2 only observed in PLP 2010 and explaining the highest PVE of 6.5% to reduce FHB severity. The markers associated with the QTL on 2A, 3A, 3B, 4B, 5A, and 6A chromosomes identified with the integrated map coincide with or are mapped nearby the markers identified using the DArT/SSR map and the high-density SNP map.

**Table 6:** Summary of Fusarium head blight (FHB) QTL detected from the 261 sets of the population using the SNP/DArT/SSR map, number of test environments (Portage la Prairie (PLP) in 2010, 2011, and 2013; Morden (MDN) in 2015, 2016, and 2017; and Brandon (BDN) in 2016 and 2017) and other traits associated with FHB QTL, markers associated with the highest LOD values, and FHB resistance allele contributing parent.

Chr.	QTL	The total number of environments analyzed	Number of environments where QTL identified	Location/year	FHB trait	Co-location with	Markers with the highest LOD	Highest LOD	Highest PVE (%)	Source of FHB resistance
2A	*Qfhb.spa-2A*	8	1	PLP13	INC		*wPt-665330/BS00041816_51*	3.3	5.6	Carberry
3A	*Qfhb.spa-3A.1*	8	1	MDN15	INC, SEV, IND	MAT	*wsnp_Ex_c10272_16842803*	5.3	9.9	AC Cadillac
	*Qfhb.spa-3A.2*	8	1	PLP13	INC		*BS00108976_51*	3.4	6.5	Carberry
3B	*Qfhb.spa-3B.1*	8	6	MDN15, MDN16, MDN17, BDN16, BDN17, PLP13	INC, SEV, IND		*BobWhite_c2453_340*	11.9	20.9	Carberry
	*Qfhb.spa-3B.2*	8	1	PLP10	SEV, IND		*Barc75/wPt-2748*	3.4	6.5	Carberry
4B	*Qfhb.spa-4B*	8	5	MDN16, MDN17, BDN17, PLP10, PLP11	INC, SEV, IND	PH, DTH, MAT	*wPt744434/Excalibur_c29141_864*	10.8	19.2	AC Cadillac
5A	*Qfhb.spa-5A.1*	8	5	MDN15, MDN17, BDN17, PLP10, PLP11	INC, SEV, IND		*BobWhite_c8266_227*	21.3	34.4	Carberry
6A	*Qfhb.spa-6A*	8	1	MDN17	SEV, IND		*wsnp_BE495143A_Ta_2_2*	3.9	7.4	AC Cadillac
6D	*Qfhb.spa-6D*	8	1	PLP10	INC, SEV	PH	*wPt-741955*	3.6	6.8	Carberry

INC, FHB incidence; SEV, severity; IND, index; PVE, phenotypic variation explained; MAT; maturity; PHEIGHT; abbreviations for test locations followed by the last two digits of experiment years.

### FHB QTL identified in the 775 DH set

The analysis by MQM on the 775 DH set revealed 17 significant QTL for FHB resistance located on 14 wheat chromosomes (**Table 7**). **Table 8** gives a list of putative genes annotated using the Chinese Spring reference near the FHB QTL identified in this study. QTL that accounted for phenotypic variations greater than 10% were considered major-effect loci, and such loci were identified on chromosomes 3B (*Qfhb.spa-3B.1*), 4B (*Qfhb.spa-4B*), and 5A (*Qfhb.spa-5A.1*). QTL that explained less than 10% of the phenotypic variations were considered minor-effect loci, and they were located on chromosomes 1A (*Qfhb.spa-1A*), 1B (*Qfhb.spa-1B*), 2A (*Qfhb.spa-2A*), 2B (*Qfhb.spa-2B*), 3A (*Qfhb.spa-3A.1* and *Qfhb.spa-3A.2*), 4A (*Qfhb.spa-4A*), 4B (*Qfhb.spa-4B*), 5A (*Qfhb.spa-5A.2, Qfhb.spa-5A.3*), 6A (*Qfhb.spa-6A*), 6B (*Qfhb.spa-6B*), 7A (*Qfhb.spa-7A*), 7B (*Qfhb.spa-7B*), and 7D (*Qfhb.spa-7D*). Carberry was the source of resistance alleles for the major-effect loci *Qfhb.spa*-*3B.1* and *Qfhb.spa-5A.1*, as well as the minor-effect loci *Qfhb.spa-1A*, *Qfhb.spa-2A*, *Qfhb.spa-3A.2*, and *Qfhb.spa-7D*. AC Cadillac contributed resistance alleles for a major effect locus at *Qfhb.spa-4B* and minor-effect loci *Qfhb.spa-1B*, *Qfhb.spa-2B*, *Qfhb.spa-3A.1*, *Qfhb.spa-4A*, *Qfhb.spa-5A*.*2*, *Qfhb.spa-5A*.*3*, *Qfhb.spa-6A*, *Qfhb.spa-6B*, *Qfhb.spa-7A*, and *Qfhb.spa-7B*. The QTL *Qfhb.spa-3B.1* greatly reduced FHB severity with an expressed PVE of 10.2% in the MDN 2015 environment, 15.6% in the MDN 2016 environment, and 11.4% in the BDN 2017 environment. The effect of the QTL on the FHB index reached 13.1% of the explained variation in the trait at MDN in 2016. *Qfhb.spa-4B* accounted for 10.5% to 21.2% of the variation explained in the FHB incidence, severity, and index across MDN and BDN environments. QTL mapping, which was performed using 336 lines carrying the reduced height gene *Rht-B1* extracted from the 775 DH set, failed to detect the *Qfhb.spa-4B* that was detected using the unpartitioned population. The third major QTL, *Qfhb.spa-5A.1*, had an effect mainly against the FHB index (PVE = 11.4%) and FHB severity (PVE = 13.1%) in MDN 2015 and the index (PVE = 11.3%) in BDN 2017. Despite having minor effects, QTL such as *Qfhb.spa-2B* and *Qfhb.spa-3A.1* influenced all three FHB components and were fairly consistent over the environments (**Table 7**). In contrast, QTL such as *Qfhb.spa-*1A, *Qfhb.spa-*1B, *Qfhb.spa-*2A, *Qfhb.spa-3A.2*, *Qfhb.spa-*6A, *Qfhb.spa-*6B, *Qfhb.spa-*7A, *Qfhb.spa-*7B, and *Qfhb.spa-*7D are expressed only in single environments.

**Table 7:** Summary of Fusarium head blight (FHB) QTL identified in the 775 sets of lines of the Carberry/AC Cadillac population, number of test environments, phenological and morphological traits co-located with FHB QTL, markers associated with the highest LOD values, and FHB resistance allele contributing parent.

Chr.	QTL	Total number of environments analyzed	Number of environments where QTL identified	Location/year	FHB trait	Co-location with	Markers with the highest LOD	Highest LOD	Highest PVE (%)	Source of FHB resistance
1A	*Qfhb.spa-1A*	5	1	MDN17	INC, SEV, IND		*Excalibur_c75270_566*	4	2.4	Carberry
1B	*Qfhb.spa-1B*	5	1	BDN17	INC, SEV, IND		*BS00070283_51*	4.5	2.6	AC Cadillac
2A	*Qfhb.spa-2A*	5	1	MDN17	INC, SEV, IND		*BS00041816_51/BS00022241_51*	4.2	2.3	Carberry
2B	*Qfhb.spa-2B*	5	5	MDN15, MDN16, MDN17, BDN16, BDN17	INC, SEV, IND	DTH	*TA002079-5951/RAC875_c6358_1091*	8	4.7	AC Cadillac
3A	*Qfhb.spa-3A.1*	5	5	MDN15, MDN16, MDN17, BDN16, BDN17	INC, SEV, IND	DTH, MAT	*RAC875_rep_c100137_586*	10.2	5.9	AC Cadillac
3A	*Qfhb.spa-3A.2*	5	1	MDN16	SEV		*BS00108976_51*	3.2	1.8	Carberry
3B	*Qfhb.spa-3B.1*	5	5	MDN15, MDN16, MDN17, BDN16, BDN17	INC, SEV, IND		*BobWhite_c2453_340/BobWhite_c2453_282*	28.6	15.6	Carberry
4A	*Qfhb.spa-4A*	5	4	MDN15, MDN17, BDN16, BDN17	INC, SEV, IND	DTH	*BobWhite_c4089_73*	6.0	3.5	AC Cadillac
4B	*Qfhb.spa-4B*	5	5	MDN15, MDN16, MDN17, BDN16, BDN17	INC, SEV, IND	MAT, PH	*BS00021984_51/wsnp_BF482960B_Ta_1_4*	40.2	21.2	AC Cadillac
5A	*Qfhb.spa-5A.1*	5	4	MDN15, MDN16, MDN17, BDN17	INC, SEV, IND	AWN	*BobWhite_c8266_227*	23.7	13.1	Carberry
5A	*Qfhb.spa-5A.2*	5	2	MDN17, BDN16	INC, SEV, IND	DTH	*wsnp_Ku_c3953_7233359*	4.8	2.8	AC Cadillac
5A	*Qfhb.spa-5A.3*	5	2	BDN16, BDN17	INC, SEV, IND		*BS00077855_51*	6.8	3.7	AC Cadillac
6A	*Qfhb.spa-6A*	5	1	BDN17	SEV		*Excalibur_rep_c105491_144*	3.5	2	AC Cadillac
6B	*Qfhb.spa-6B*	5	1	MDN16	INC		*IAAV3657*	2.7	1.3	AC Cadillac
7A	*Qfhb.spa-7A*	5	2	MDN17, BDN17	INC, SEV, IND		*Tdurum_contig54832_139*	3.4	2	AC Cadillac
7B	*Qfhb.spa-7B*	5	1	BDN17	INC		*BS00064783_51*	3.3	1.9	AC Cadillac
7D	*Qfhb.spa-7D*	5	1	BDN16	INC, SEV, IND	PH, MAT	*Kukri_c35508_426*	8.3	3.9	Carberry

INC, FHB incidence; SEV, severity; IND, index; PVE, phenotypic variation explained; MAT, maturity; PH, plant height; abbreviations for test locations followed by the last two digits of experiment years.

**Table 8:** List of annotated genes close to markers associated with Fusarium head blight resistance quantitative trait loci (QTL) detected in the Carberry/AC Cadillac population.

Chr.	FHB QTL	Markers with the highest LOD	IWGSC Chinese Spring Wheat Refseq. V.1						
			Marker interval (Bp)	Closest gene^a^	Gene interval (Bp)	Strand	Confidence	Gene type	Gene description
1A	*Qfhb.spa-1A*	*Excalibur_c75270_566*	41901048–41901148	*TraesCS1A02G061100*	41900965–41901385	+	H	Protein coding	n/a
1B	*Qfhb.spa-1B*	*BS00070283_51*	88004411–88004511	*TraesCS1B02G089100*	88002205–88005357	−	H	Protein coding	n/a
2A	*Qfhb.spa-2A*	*wPt-9320*	60709058–60709473	*TraesCS2A02G108600*	60710672–60711807	−	H	Protein coding–peroxidase	Peroxidase
2A	*Qfhb.spa-2A*	*wPt-0873*	700960766–700961127	*TraesCS2A02G451300*	700954858–700962731	−	H	Protein coding	n/a
2A	*Qfhb.spa-2A*	*BS00041816_51*	611316318–611316418	*TraesCS2A02G367700*	611315173–611317329	−	H	Protein coding	n/a
2A	*Qfhb.spa-2A*	*BS00022241_51*	663328917–663329017	*TraesCS2A02G407700*	663327624–663330251	−	H	Protein coding	n/a
2B	*Qfhb.spa-2B*	*TA002079-5951*	45788663–45788716	*TraesCS2B02G082500*	45787000–45801095	−	H	Protein coding	Plastid acetyl-CoA carboxylase
2B	*Qfhb.spa-2B*	*RAC875_c6358_1091*	47839660–47839760	*TraesCS2B02G086200*	47839515–47840915	−	H	Protein coding	n/a
3A	*Qfhb.spa-3A.1*	*wsnp_Ex_c10272_16842803*	n/a		–				
3A	*Qfhb.spa-3A.2*	*BS00108976_51*	730177975–730178075	*TraesCS3A02G509800*	730177399–730178426	−	H	Protein coding	n/a
3A	*Qfhb.spa-3A.1*	*RAC875_rep_c100137_586*	156268313–156268413	*TraesCS3A02G158600*	156267711–156271635	−	H	Protein coding	n/a
3B	*Qfhb.spa-3B.1*	*wPt-744251*	308120612–308120933	*TraesCSU02G207600*	308121181–308123171	−	H	Protein coding	n/a
3B	*Qfhb.spa-3B.1*	*wPt-2757*	35312963–35313825	*TraesCSU02G044100*	35310604–35314593	−	H	Protein coding	n/a
3B	*Qfhb.spa-3B.1*	*BobWhite_c2453_340*	6252144–6252244	*TraesCS3B02G014900*	6250392–6253500	−	H	Protein coding	n/a
3B	*Qfhb.spa-3B.1*	*BobWhite_c2453_282*	6252202–6252302	*TraesCS3B02G014900*	6250392–6253500	−	H	Protein coding	n/a
3B	*Qfhb.spa-3B.2*	*Barc75*	3395394–3395500	*TraesCS3B02G006500*	3407143–3409183	+	H	Protein coding	n/a
3B	*Qfhb.spa-3B.2*	*RAC875_c101155_64*	3665640–3665740	*TraesCS3B02G007200*	3665420–3667378	−	H	Protein coding	n/a
3B	*Qfhb.spa-3B.2*	*wPt-2748*	6172276–6173048	*TraesCS3B02G015200LC*	6164922–6169177	+	L	Protein coding	n/a
4A	*Qfhb.spa-4A*	*BobWhite_c4089_73*	583968986–583969085	*TraesCS4A02G273100*	583968986–583972215	−	H	Protein coding	n/a
4B	*Qfhb.spa-4B*	*wPt-744434.1*	350195761–350196151	*TraesCSU02G496700LC*	350235301–350236704	−	L	Protein coding	n/a
4B	*Qfhb.spa-4B*	*wPt-744434.2*	297069069–297069458	*TraesCSU02G383800LC*	297159329–297160021	−	L	Protein coding	n/a
4B	*Qfhb.spa-4B*	*Xwmc617*	15769693–15769907	*TraesCS4B02G021900*	15759954–15769797	−	H	Protein coding	n/a
4B	*Qfhb.spa-4B*	*BS00021984_51*	35520722–35520822	*TraesCS4B02G047900*	35520142–35526324	−	H	Protein coding	NADH dehydrogenase [ubiquinone] iron-sulfur protein 1, mitochondrial
4B	*Qfhb.spa-4B*	*wsnp_BF482960B_Ta_1_4*	28954488–28954608	*TraesCS4B02G042300*	28949360–28960436	+	H	Protein coding	n/a
4B	*Qfhb.spa-4B*	*Excalibur_c29141_864*	281734952–281735052	*TraesCS4B02G155800*	281732350–281735921	−	H	Protein coding	n/a
5A	*Qfhb.spa-5A.1*	*Xgwm595*	680066700–680066886	*TraesCS5A02G690000LC*	680075048–680078012	−	L	Protein coding	n/a
5A	*Qfhb.spa-5A.1*	*wPt-1038*	671393599–671394085	*TraesCS5A02G506500*	671390845–671393558	+	H	Protein coding	Cyclin-dependent kinase inhibitor
5A	*Qfhb.spa-5A.1*	*BobWhite_c8266_227*	698508129–698508213	*TraesCS5A02G542600*	698507247–698511217	+	H	Protein coding	n/a
5A	*Qfhb.spa-5A.1*	*BS00022864_51*	700371752–700371852	*TraesCS5A02G545100*	700359844–700372053	+	H	Protein coding	n/a
5A	*Qfhb.spa-5A.2*	*Kukri_c86812_193*	663374163–663374263	*TraesCS5A02G495300*	663371090–663374633	+	H	Protein coding	Cytochrome *c*-type biogenesis protein
5A	*Qfhb.spa-5A.2*	*Excalibur_c60150_107*	665473709–665473805	*TraesCS5A02G498100*	665470983–665478767	−	H	Protein coding	n/a
5A	*Qfhb.spa-5A.2*	*wsnp_Ku_c3953_7233359*	n/a		–				n/a
5A	*Qfhb.spa-5A.3*	*BS00077855_51*	559506234–559506334	*TraesCS5A02G357200*	559505557–559507785	+	H	Protein coding	n/a
6A	*Qfhb.spa-6A*	*wsnp_BE495143A_Ta_2_2*	580198764–580198884	*TraesCS6A02G347800*	580196254–580203070	+	H	Protein coding	Diacylglycerol kinase
6A	*Qfhb.spa-6A*	*Excalibur_rep_c105491_144*	389983417–389983517	*TraesCS6D02G282800*	389980868–389984718	+	H	Protein coding	Alpha-galactosidase
6B	*Qfhb.spa-6B*	*IAAV3657.1*	705562896–705563096	*TraesCS6B02G440900*	705561741–705565349	+	H	Protein coding	n/a
6B	*Qfhb.spa-6B*	*IAAV3657.2*	609791181–609791379	*TraesCS6A02G401000*	609790120–609797040	+	H	Protein coding	n/a
6D	*Qfhb.spa-6D*	*wPt-741955*	5153213–5153642	*TraesCS6D02G012900*	5149646–5153289	−	H	Protein coding	n/a
7A	*Qfhb.spa-7A*	*Tdurum_contig54832_139*	700677526–700677626	*TraesCS7A02G514600*	700670526–700677759	+	H	Protein coding	n/a
7B	*Qfhb.spa-7B*	*BS00064783_51*	616502245–616502345	*TraesCS7D02G517700*	616502120–616504728	−	H	Protein coding	n/a
7D	*Qfhb.spa-7D*	*Kukri_c35508_426*	n/a	–	–	–	–	–	n/a

n/a, information not available.

a Source: https://plants.ensembl.org/Triticum_aestivum/Tools/Blast.

### QTL for phenological and morphological traits not co-located with FHB

The QTL for the phenological and morphological traits including plant height, days to heading, and days to maturity that were not associated with the FHB resistance are summarized in **Table 9**. QTL for plant height not co-located with FHB were detected in different environments on chromosomes 3A (*Qph.spa-3A*), 5A (*Qph.spa-5A*), 6D (*Qph.spa-6D*), and 7D (*Qph.spa-7D*). Carberry conditioned alleles for reduced height at *Qph.spa-3A* and *Qph.spa-5A*, whereas AC Cadillac conditioned alleles for reduced height at *Qph.spa-6D* and *Qph.spa-7D*. This reduced-height QTL is expressed in specific environments. QTL for days to heading and not co-located with FHB QTL were detected on chromosomes 1A (*Qdth.spa-1A*), 4B (*Qdth.spa-4B*), 4D (*Qdth.spa-4D*), 5A (*Qdth.spa-5A*), and 7D (*Qdth.spa-7D*) (**Table 9**). Carberry contributed alleles for early heading on chromosomes 1A, 4D, and 5A, whereas AC Cadillac contributed alleles for early heading on 4B and 7D. QTL on chromosomes 4B, 5A, and 7D was consistently revealed when the QTL mapping was carried out using the DArT/SSR map, the high-density SNP map, and the map comprising all the markers. Four QTL for days to maturity and not co-localized with FHB were detected on chromosomes 2B (*Qmat.spa-2B*), 3A (*Qmat.spa-3A*), 4D (*Qmat.spa-4D*), and 6B (*Qmat.spa-6B*) using different genetic maps and population sets (**Table 9**). Alleles for early maturity were contributed by Carberry on 2B, 3A, and 4D, whereas AC Cadillac was the source of alleles for early maturity on 6B.

**Table 9:** Summary of phenological and morphological QTL that were not associated with FHB QTL detected in the 261 and 775 sets of the population, markers associated with the highest LOD values, and QTL contributing parent.

Chr.	QTL	Detection in the 261 set	Detection in the 775 set	Markers with the highest LOD	Highest PVE (%)	QTL contributors
1A	*Qdth.spa-1A*	+	−	*wPt-731807/WwPt-730148*	4.8	Carberry
2B	*Qmat.spa-2B*	−	+	*Excalibur_c75270_566*	2.3	Carberry
3A	*Qmat.spa-3A*	+	−	*rPt-9057/rPt-2940*	7.8	Carberry
3A	*Qph.spa-3A*	−	+	*wsnp_Ku_c3286_6111264*	2.0	Carberry
4B	*Qdth.spa-4B*	+	+	*wPt-5303/wPt-1849 (SS)*	5.2	AC Cadillac
				*wsnp_Ex_c19844_28854305 (LS)*	1.9	
4D	*Qdth.spa-4D*	+	+	*wPt-0941/wPt-5630 (SS)*	6.2	Carberry
				*wsnp_Ex_rep_c79748_75305162/BS00022436_51 (LS)*	1.9	
	*Qmat.spa-4D*	+	+	*wPt-671760/Xwmc52 (SS)*	5.4	Carberry
				*RAC875_c40619_130 (LS)*	2	
5A	*Qdth.spa-5A*	+	−	*Xcfd39/Xcfa2141*	9.1	Carberry
	*Qph.spa-5A*	+	−	*wPt-4262/wPt-8226*	8.2	Carberry
6B	*Qmat.spa-6B*	−	+	*Excalibur_c63243_434/Excalibur_c91980_108*	2.3	AC Cadillac
6D	*Qph.spa-6D*	+	−	*wPt-741955/wPt-2864*	3.7	AC Cadillac
7D	*Qdth.spa-7D*	+	+	*wPt-744846/wPt-745036 (SS)*	12.5	AC Cadillac
				*Kukri_c23468_590 (LS)*	3.0	
	*Qph.spa-7D*	+	−	*wPt-1859/wPt-665936*	4.1	AC Cadillac

PVE%, phenotypic variance explained; DTH, days to heading; MAT, maturity; PH, plant height; “+” QTL detected; “−” QTL not detected; SS smaller set of the population; LS, larger set of the population.

### Co-location of FHB QTL with phenological and morphological trait QTL

QTL coincident for FHB resistance and different phenological and morphological traits are given in **Tables 5**–**7**. The most prominent QTL for reduced plant height on chromosome 4B overlapped with FHB resistance, days to heading, and days to maturity. Plant height loci also overlapped with FHB resistance QTL on chromosomes 6D and 7D, where the reduced height loci were contributed by AC Cadillac and the FHB resistance loci by Carberry. Early heading loci were mapped on chromosomes 4B, 4A, and 5A2, overlapping with FHB resistance loci. While the FHB resistance loci were contributed by AC Cadillac, the early heading loci were contributed by Carberry. For early maturity, loci overlapped with FHB resistance on chromosomes 3A1, 4B, and 7D. FHB resistance on 3A1 and 4B was contributed by AC Cadillac, while the early maturity was contributed by Carberry. On chromosome 7D, the early maturity locus was contributed by AC Cadillac, while FHB resistance was contributed by Carberry.

A major QTL explaining 92% of the phenotypic variation was identified on chromosome 5A controlling the presence of awns, with the allele for the presence of awns contributed by Carberry associated with *Qfhb.spa-5A.1* (**Table 7**).

## Discussion

The frequency distribution patterns of the Carberry/AC Cadillac progeny lines for disease incidence, severity, and the FHB index in response to the FHB infections in different environments suggest the quantitative nature of the resistance, in which several minor-effect genes are involved. The identification of the FHB resistance QTL on only five chromosomes with the different genetic maps generated on the 261 set of lines compared with the detection of QTL on 14 chromosomes when the high-density map developed using the 775 DH set of lines was used indicates the power of using larger populations and genetic maps with higher marker density to disclose new chromosome loci controlling the expression of the traits of interest. The power of using a large population and high-density map was also exhibited in the detection of the majority of the QTL with minor effects, as they were only detected using the larger set, suggesting that the expansion of the population size allowed the identification of additional resistance loci with minor effects (Charmet, 2000).

The inconsistent QTL expression that was observed across environments in the present study is irrespective of the population size. For example, the QTL *Qfhb.spa-3B.1*, *Qfhb.spa-4B*, and *Qfhb.spa-5A1* were stable across environments as compared with *Qdth.spa-1A*, *Qfhb.spa-1B*, *Qfhb.spa-6B*, *Qfhb.spa-6D*, and *Qfhb.spa-7D*, which were identified in a single environment. The genotype-by-environment interaction had an influence on the expression of the different QTL, which is consistent with the low-to-moderate correlations over environments and heritability of FHB traits. It is well-known and reported that breeding for FHB resistance is complex due to the large effect of the environment, complex inheritance, and significant genotype-by-environment interaction (Buerstmayr et al., 2012; Steiner et al., 2017). Buerstmayr et al. (2009) stressed the importance of increasing the number of environments for FHB evaluation to determine and estimate the stability of a QTL. Our study demonstrated the effect of population size, number of environments, and map density on the power of QTL detection through the evaluation of the small set across eight environments using the three different genetic maps. This small set revealed environment-specific QTL such as *Qfhb.spa-3A.1.* When we analyzed FHB data collected from additional environments, additional QTL with minor effects, such as *Qfhb.spa-6D*, were detected when the initial DArT/SSR genetic map was complemented with SNP markers, confirming the findings reported by Charmet (2000) and Buerstmayr et al. (2009).

The Carberry-derived QTL, *Qfhb.spa-3B.1*, is stable, as it was detected in both sets of the population in all the tested environments with the exception of PLP 2013. The *Qfhb.spa-3B.1* genomic region was located in the same interval as *Fhb1*, previously reported by Cuthbert et al. (2006). The variation observed in the response of the *Qfhb.spa-3B.1* to the FHB infection across environments indicates the expression of this QTL is significantly influenced by environmental variations. The greater effect of *Qfhb.spa-3B.1* on disease severity compared with its effect on the disease incidence indicates the QTL is useful in preventing the spread and further invasion of the wheat spikes with fungal spores. *Fhb1* is the largest-effect resistance QTL in wheat, and its effectiveness in providing resistance to disease severity is frequently reported (Waldron et al., 1999; Anderson et al., 2001; Buerstmayr et al., 2002; Buerstmayr et al., 2003; Cuthbert et al., 2006). Our results provide evidence for consistent transmission and expression of the *Fhb1* locus through Carberry as a descendant of Alsen, which is derived from the three-way cross ND674//ND2710/ND688, where the line ND2710 is derived from a cross involving Sumai3 (Bokore et al., 2017).

The present study also showed that the *Qfhb.spa-3B.1* was not associated with plant height, which is in agreement with other studies (Buerstmayr et al., 2009; Giancaspro et al., 2016; Prat et al., 2017). The nonassociation of *Qfhb.spa-3B.1* QTL with plant height indicates the gene could be introgressed into other lines without affecting plant height. The semi-dwarf and early maturing variety Carberry was the source of the resistance allele for the *Qfhb.spa-3B.1*, making the selection of short plants possible. The reasons why Carberry was more resistant than late and tall cultivars such as AAC Cadillac could be because Carberry has major-effect QTL that are not associated with days to heading or plant height, such as *Qfhb.spa-3B.1* (*Fhb1*) and *Qfhb.spa-5A.1*, along with other QTL/genes such as *Qfhb.spa-2A.2* and *Qfhb.spa-7D*. Because of this, Carberry and its derivatives have been used in several crosses to enhance FHB resistance in the Canadian Spring wheat-adapted germplasm. Recently, Prat et al., 2017 successfully validated the introgression of *Fhb1* from hexaploid wheat into durum wheat, which resulted in improved FHB resistance. The authors reported that the negative effect of the semi-dwarfing *Rht-B1* locus on FHB resistance was largely compensated in lines carrying *Fhb1*.

The *Qfhb.spa-4B* has been identified in both the 261 and 775 DH set but showed variable expression on FHB infection, as indicated by the amount of phenotypic variance explained. *Qfhb.spa-4B* attributed to AC Cadillac coincides with the reduced-height QTL corresponding to the *Rht-B1* gene, where the Carberry allele conditioned the short plant height type. The presence of the *Rht-B1* gene in Carberry was previously reported by Pandey et al. (2015). Numerous studies have reported increased susceptibility to the FHB disease in short genotypes having the *Rht1* gene (Paillard et al., 2004; Steiner et al., 2004, Schmolke et al., 2008; Srinivasachary et al., 2008a, Szabó-Hevér et al., 2014). Miedaner and Voss (2008) studied the effect of dwarfing genes on FHB resistance in two sets of near-isogenic wheat lines, and they found that *Rht* genes, including *Rht-B1d*, increased FHB ratings significantly. This is further supported by our current research, in which no QTL for FHB resistance on 4B was identified when we grouped lines carrying the *Rht-B1* gene and performed QTL analysis on the two pools. Although resistance might be attributed to disease escape in the tall genotypes, Nicholson et al. (2016) suggested the direct role of plant height hormone gibberellic acid in the resistance of wheat to FHB, supporting the involvement in physiological resistance rather than morphological resistance. This pleiotropic effect of an FHB resistance gene and a plant height gene may exist in some genotypes. In addition, a close linkage between loci conferring FHB resistance and plant height may exist, as suggested by Srinivasachary et al. (2009) and Sari et al. (2018). The low-to-moderate correlations between FHB traits and height were suggestive of an association between height and FHB level, but other factors could also be involved.

The high correlation observed between plant height and FHB incidence compared to the correlation between plant height and FHB severity in the present study suggests that the taller plants escaped the first infection due to the reduced dispersal of ground inoculum to the spike, which agrees with other studies (Mesterhazy, 1995; Hilton et al., 1999). Several studies reported that resistance to FHB severity is less affected by plant height than resistance to FHB incidence (Steiner et al., 2004; Srinivasachary et al., 2008a; Srinivasachary et al., 2008b; Srinivasachary et al., 2009; Lu et al., 2011). Yan et al. (2011) demonstrated that the negative effect of most semi-dwarf alleles, including *Rht-B1*, on FHB incidence resistance disappeared when semi-dwarf lines that were isogenic for height were physically raised so that their spikes were the same height as those of their taller counterparts. The semi-dwarf gene *Rht‐B1b* is associated with reduced anther extrusion, leading to an increased FHB incidence (He et al., 2016), and anther extrusion is related to susceptibility (Lu et al., 2013; Buerstmayr and Buerstmayr, 2015; He et al., 2016). Similarly, Lu et al. (2013) reported that FHB severity was negatively correlated with both anther extrusion and plant height in a recombinant inbred line population evaluated in two countries, Norway and China. In a study conducted in Austria, Buerstmayr and Buerstmayr (2016) found that recombinant inbred lines with a low proportion of retained anthers after flowering and tall plants were much less prone to disease, suggesting anther retention is highly associated with FHB susceptibility. Thus, anther extrusion could be used as an indirect selection criterion in resistance breeding.

The *Qfhb.spa-5A.1* from Carberry must be a different gene than *Qfhb.spa-5A.2* and *Qfhb.spa-5A.3* because the source of the latter two QTL is AC Cadillac. The distinctness between the QTL is supported by the results of a BLAST search in the Chinese spring IWGSC RefSeq v1.0 that placed *Qfhb.spa-5A.1* at 41.3 Mbp away from the *Qfhb.spa-5A.2* and 139 Mbp from *Qfhb.spa-5A.3* QTL. Additionally, detecting three QTL associated with FHB resistance on chromosome 5A is not unusual. The literature notes numerous FHB QTL on 5A from several germplasms. The QTL on chromosome 5A derived from Sumai3 (*Qfhs.ifa-5A*) (Buerstmayr et al., 2002, 2003) is among the frequently studied FHB resistance QTL and is validated either individually or in combination with other QTL (Chen et al., 2006; Chrpova et al., 2011; Bokore et al., 2017; Kang et al., 2011; McCartney et al., 2007; von der Ohe et al., 2010; Salameh et al., 2011; Suzuki et al., 2012; Tamburic-Ilincic, 2012). *Qfhs.ifa-5A* has recently been fine-mapped and separated into a major QTL that maps across the centromere (*Qfhs.ifa-5Ac*) and a minor QTL positioned in the distal half of 5AS (*Qfhs.ifa-5AS*) (Steiner et al., 2019). *Fhb5* is another well-documented QTL for FHB resistance on chromosome 5A derived from the Chinese wheat landrace Wangshuibai (Xue et al., 2011). The position of *Fhb5* has been further revised to a 0.09-cM interval very close to the centromere that partially overlaps the previously described *Qfhs.ifa-5Ac* by Steiner et al. (2019), suggesting a common resistance gene for the two FHB QTL. Carberry’s pedigree includes Sumai3, and it appears, based on the location of *Qfhb.spa.5A.1* being similar to *Qfhs.ifa-5A* and *Fhb5*, that the genomic region controlling FHB resistance from Sumai3 on chromosome 5A has been transmitted to Carberry.

The consistent expression of the *Qfhb.spa-5A.1* in both the 261 and 775 DH set with, at times, a strong phenotypic expression with the variance explained ranging up to 16.3% makes it an appealing locus for breeding. Marker *Xgwm595* was identified to be associated with *Qfhb.spa_5A.1* in the 261 set, while in the 775 DH set, *Qfhb.spa_5A.1* mapped between SNP markers *BobWhite_c8266_227* and *BS00022864_51*. In the Wheat Chinese Spring RefSeq. V1.0 genome assembly, *Xgwm595* was about 18 Mbp distant from *BobWhite_c8266_227*, whereas it was 20 Mbp from *BS00022864_51*. *Xgwm595* was reported to be associated with the FHB resistance QTL located on chromosome 5AL in a Renan × Recital winter wheat population (Gervais et al., 2003). The co-location of the *Qfhb.spa-5A.1* with the QTL for awnedness in Carberry may suggest the usefulness of the trait as a morphological marker in breeding for FHB resistance. The 5A Carberry’s FHB resistance and awnedness QTL marker, *BobWhite_c8266_227*, has been reported by MacKay et al. (2014) as a highly diagnostic marker for the morphological trait “awn presence/absence” of the *B1* gene for awnedness, indicating the presence of this gene in Carberry. The *B1* gene has been used as a physical marker, located distal to the major genes controlling vernalization requirement (*VRN-A1*) and the spelled head type (*Q*) on 5AL (Kato et al., 1998). The *Q* locus has the role of conditioning the threshing habit, and it pleiotropically affects many other important traits such as plant height, spike shape, ear compactness, and glume shape (Faris and Gill, 2002; Simons et al., 2006). He et al. (2016) identified QTL for FDK resistance, tenacity of glumes, and anther extrusion at the Q locus. In our population, it is possible that part of the increased FHB resistance may be due to morphological traits such as awnedness, which agrees with the results reported by Gervais et al. (2003). The linkage between FHB resistance and the *B1* gene for awnedness was first reported by Snijders (1990), who stated that the presence of awns and FHB resistance are genetically linked, suggesting awned progenies of a resistant awned parent should be selected to indirectly select for partial resistance to FHB.

That AC Cadillac’s FHB resistance at *Qfhb.spa-5A.2* co-located with the days to heading QTL, where the delayed days to heading allele were contributed by AC Cadillac, indicated the possibility of a disease escape attributed to lateness rather than physiological resistance at this QTL. The possibility of passive resistance resulting from disease escape attributed to delayed heading has been reported in several studies (Miedaner et al., 2006; Klahr et al., 2007; Wilde et al., 2007). The other AC Cadillac QTL on chromosome 5A, *Qfhb.spa-5A.3*, was not associated with days to heading or plant height, suggesting a real allele for FHB resistance was in action. The failure of this QTL to be detected in the 261 set confirms once again the power of increasing population size on the detection of QTL with minor effects.

Despite its minor effect, the *Qfhb.spa-2B* QTL was consistent across environments for the 775 DH set. A literature review by Buerstmayr et al. (2009) indicated that other studies revealed QTL for FHB resistance on chromosome 2B in several mapping populations, supporting the idea that the QTL effect is real. Chromosome 2B was also reported to harbor several QTL for morphological and developmental traits. The locus *Xfbb121-2B* mapped on chromosome 2B was reported to be associated with photoperiod response and heading time in the cross Courtot with Chinese Spring (Sourdille et al., 2000). Gervais et al. (2003) reported an overlap of FHB resistance QTL with plant height QTL and flowering date QTL on chromosome 2B in the winter wheat cross Renan by Récital. Furthermore, Gilsinger et al. (2005) reported a QTL for resistance to FHB that shared a position with a narrow flowering opening time on chromosome 2B. The association of a reduced FHB infection with a late heading that was observed in the 775 DH set indicates these two traits are negatively correlated. Studies have shown that the repulsion relationship between days to heading QTL and FHB QTL is likely due to close linkage rather than pleiotropy (Nduulu et al., 2007; Massman et al., 2011). Nevertheless, it may not be difficult to select early heading lines, considering the moderate-to-low correlation coefficients and as evidenced by Carberry being earlier to head than AC Cadillac.

In the 775 DH set, MQM detected two QTL conferring resistance to FHB on chromosome 3A, *Qfhb.spa-3A.1* derived from AC Cadillac and *Qfhb.spa-3A.2* from Carberry. The first QTL, *Qfhb.spa-3A.1*, could be considered stable as it is expressed across most environments and associated with resistance to FHB incidence, severity, and index. In contrast, the second QTL, *Qfhb.spa-3A.2*, could be considered unstable as it only appeared in a single environment associated with FHB severity. Although the relationship with the QTL identified in the current study remains to be unclear, chromosome 3A has been reported to carry FHB resistance in different wheat genotypes of Chinese, South American, and European genetic backgrounds (Gervais et al., 2003; Buerstmayr et al., 2009). The overlap of *Qfhb.spa-3A.1* with the heading date and maturity similar to the 4B QTL could indicate that days to heading and maturity contributed to passive resistance as a form of disease escape or that linkages may exist between genes for FHB resistance and days to heading and maturity or, alternatively, from pleiotropic effects among genes. However, weak-to-moderate correlations of days to heading and maturity with FHB resistance suggest that factors other than just days to heading and maturity affect resistance. Despite its inconsistency across environments, Carberry’s QTL *Qfhb.spa-3A.2* was independent of any morphological or developmental traits, making it suitable for use in MAS of FHB resistance gene stacking.

The co-location of FHB resistance with days to heading QTL identified on chromosome 4A and maturity QTL on chromosome 7D suggests that in addition to the phenotypic screening for FHB resistance, careful evaluation for morphological and developmental traits must be undertaken. With morphological and developmental traits that have an influence on modulating FHB reaction directly or through linkage with FHB resistance per se, it is very important to break the linkages and select alternate morphological and developmental loci that are independent of resistance that complement pleiotropic resistance loci. For example, if the AC Cadillac’s *Qfhb.spa-5A.2* locus identified in the present study is pleiotropic for late maturity and physiological FHB resistance, other loci will need to be selected for early maturity that are unrelated to resistance if earliness is a desirable trait. Consequently, it is important to stratify breeding lines for days to heading and plant height when evaluating them for FHB resistance.

Most of the other minor FHB QTL on 1A, 1B, 2A, 6A, 6B, 7A, and 7B can be assigned to known QTL clusters based on shared markers or map position comparison (Buerstmayr et al., 2009; Liu et al., 2009; Lu et al., 2013). The absence of association of these minor QTL with any of the agro-morphological or developmental traits studied suggests these QTL can be stacked without being affected by any of the agro-morphological traits studied. These QTL should be confirmed through testing in more environments prior to being utilized in marker-assisted selection. In contrast, we identified QTL for days to maturity on each of chromosomes 2B, 4D, and 6B and plant height on chromosome 7D that were not associated with FHB resistance. These loci could be deployed in combination with FHB resistance loci associated with negative effects as a balance to other traits’ effects and achieve new wheat cultivars with an improved level of FHB resistance, desirable plant height, early heading, and maturity.

Several genes annotated using the Chinese Spring wheat genome reference shared common genomic regions with the FHB QTL identified in the current study (**Table 8**). However, the functionality of the majority of these annotated genes is not known. For example, the function of three putative genes, *TraesCSU02G207600*, *TraesCSU02G044100*, and *TraesCS3B02G014900*, located in the *Qfhb.spa-3B.1* region, was not characterized, suggesting further study to understand the relationship of these genes with the *Qfhb.spa-3B.1*. In the *Qfhb.spa-4B* region, an NADH dehydrogenase iron–sulfur protein 1 gene, *TraesCS4B02G047900*, was located. According to Xu et al. (2019), the NADH protein family could decrease DON biosynthesis of *F. asiaticum* by inhibiting glycolysis and the tricarboxylic acid cycle in wheat. A cyclin-dependent kinase (CDK) inhibitor gene, *TraesCS5A02G506500*, was located in the *Qfhb.spa-5A.1* region. In a study by Hamdoun et al. (2016), the CDK inhibitor played a regulatory defense role against bacterial infection in a model plant, *Arabidopsis* (*Arabidopsis thaliana*). Similarly, Cao et al. (2016) reported that CDK could regulate the expression of various genes important for growth, differentiation, and pathogenesis in *F. graminearum*. In rice, the cyclin-dependent protein kinase inhibitor is differentially expressed in response to powdery mildew disease (Marone et al., 2013). A cytochrome *c*-type biogenesis protein, *TraesCS5A02G495300*, was found in the *Qfhb.spa-5A.2* region. Cytochrome *c*-type biogenesis protein has been reported to protect black rot disease caused by phytopathogenic *Xanthomonas campestris* pv. *campestris* from cruciferous crops (Chen et al., 2017); however, the effect of the gene on FHB requires further research. Two proteins, diacylglycerol kinase and alpha-galactosidase, were located near the FHB QTL *Qfhb.spa-6A.* The role of diacylglycerol kinase (DGK) in biotic stress resistance, including FHB has been reported in different studies (Ding et al., 2011; Kage et al., 2017). In conclusion, the main purpose of our study was to identify and map QTL conferring resistance to FHB and investigate the relationship between the identified FHB QTL and morphological and developmental traits. A smaller set of the Carberry/AC Cadillac DH population comprising 261 lines was initially developed that was selected for reduced height and maturity and used to map FHB QTL using DArT and SSR markers. With the advent of next-generation sequencing technology, a larger number of SNP markers became available. The original population of 811 lines was subsequently phenotyped, and 775 lines were genotyped with the Illumina iSelect 90K wheat chip to generate a map with a total of 6,806 SNP markers. QTL mapping benefited from the high-density SNP map with the identification of QTL at higher precision and resolution. Despite the inherently variable environment for field evaluation of FHB reaction, stable QTL that varied in the consistency of phenotypic contribution were identified over the environments. The level of resolution provided by the larger mapping population allowed the detection of additional QTL with a minor effect. We detected a large effect of the FHB resistance QTL on chromosomes 3B, 4B, and 5A in both sets of the population, along with many other minor effect loci. FHB resistance QTL was co-localized with plant height QTL on chromosomes 4B, 6D, and 7D; days to heading on 2B, 3A, 4A, 4B, and 5A; and maturity on 3A, 4B, and 7D, with some of the associations found to be undesirable in trait improvement. However, the development of FHB-resistant cultivars is achievable with desirable plant height, days to heading, or maturity by selecting desirable morphological and developmental traits based on the loci that are not associated with FHB resistance identified in our study. The SNP markers associated with the QTL identified can be converted to KASP markers and used for MAS to improve FHB resistance through gene stacking.

## Data availability statement

The original contributions presented in the study are included in the article/supplementary material. Further inquiries can be directed to the corresponding authors.

## Author contributions

RK, RC, and SB conceived, designed, and supervised the study. RK and RC developed the mapping population, and YR and RC contributed to the seed increase and maintenance of the population. SB, AS, RC, RK, FB, MH, SK, and AB performed the field trials and FHB phenotyping. BM, AN, and CP performed genotyping and genetic data mining. SB and AS performed genetic mapping. SB analyzed the data, interpreted results and drafted the manuscript. SB, FB, RK, RD, and BM revised the manuscript. All authors reviewed the manuscript. All authors contributed to the article and approved the submitted version.
